# Contour Analysis of Three-Dimensional Peri-Implant Mucosal Model as an Endpoint Analysis of Photofunctionalization Effects on Implant Abutment Materials

**DOI:** 10.3390/ma16165529

**Published:** 2023-08-09

**Authors:** Masfueh Razali, Wen Lin Chai, Ros Anita Omar, Wei Cheong Ngeow

**Affiliations:** 1 Department of Restorative Dentistry, Faculty of Dentistry, Universiti Kebangsaan Malaysia, Kuala Lumpur 50300, Malaysia; 2 Department of Restorative Dentistry, Faculty of Dentistry, University of Malaya, Kuala Lumpur 50603, Malaysia; chaiwl@um.edu.my (W.L.C.); anitaomar@um.edu.my (R.A.O.); 3 Department of Oral & Maxillofacial Clinical Sciences, Faculty of Dentistry, University of Malaya, Kuala Lumpur 50603, Malaysia

**Keywords:** contour analysis, implant–soft tissue interface, photofunctionalization, three-dimensional oral mucosal model, ultraviolet, zirconia abutments

## Abstract

Introduction: The objective of this study was to examine the effect of photofunctionalization on the soft-tissue contour formed at the interface of various abutment materials using end-point analyses obtained from the three-dimensional oral mucosal model (3D-OMMs). Methods: Commercially pure titanium (CPTi), alumina-toughened zirconia (ATZ), and yttria-stabilized zirconia (YSZ) made into discs shapes were classified into two groups: UV-treated (PTx) and non-treated (NTx). The materials in PTx groups were exposed to UV light for 12 min. Human gingival fibroblasts and TR146 epithelial cell lines co-cultured on the acellular dermal membrane were used to construct the 3D-OMM. After 4 days of culture, the discs were inserted into the holes prepared within the membrane of 3D-OMMs. The contour formed by the tissue was evaluated after 14 days of culture. Results: The UV treatment of abutment materials resulted in the formation of more non-pocket-tissue types among the PTx group (*p* = 0.002). Of all materials tested, soft tissue contour around YSZ showed higher scores for the non-pocket type in both non- and UV-treated groups. Conclusions: The non-pocket type of tissue attachment was frequently found in all surfaces modified by photofunctionalization, particularly zirconia. The 3D-OMM can be used to evaluate the biological endpoints of implant surface modifications.

## 1. Introduction

A natural tooth and peri-implant area have similar surrounding soft tissue. The interface in which the soft tissue around implants adheres to the implant abutment represents the major difference in comparison to natural dentition [1,2,3]. The peri-implant mucosa is a stable structure that corresponds to the dentogingival area of the natural teeth. The interface consists of both epithelium and connective tissue attachments. The dynamic between the soft tissue and the implant abutment is a biological process governed by the surface characteristics of the abutment itself, such as surface chemistry and topography [4,5,6]. According to Canullo et al. [7], they demonstrated that modifications of abutment could alter and improve epithelial cells and fibroblast adhesion, thus reducing the ‘pocketing’ surrounding the transmucosal region. They described a close contact of junctional epithelium, well-vascularized subepithelial CT with collagen fibres, and a more mature CT organization at the chemically modified titanium abutment interface, as shown by studies included in their meta-analysis.

It is believed that the non-pocket mucosa formed at the implant tissue interface may perform better sealing than the pocket-type mucosa [5,8]. There are several documented advantages of subjecting the transmucosal area to surface modification, such as preserving the crystal bone, improving soft tissue attachment, minimizing the adhesion of bacterial biofilms, and facilitating strong binding between the surrounding soft tissue and the implant abutment [9,10,11]. Researchers have developed an exceptional interest in exploring the impact of the implant’s soft tissue attachment, particularly by modifying the chemistry and surface topography of implant abutments to enhance mucointegration [4,7,12,13]. As expected, surface chemistry not only influences bone integration but also plays a role in soft tissue integration [13]. The surface chemistry of an implant abutment can be carried out via photofunctionalization. The influence of photofunctionalization on osseointegration has been demonstrated by many [14,15,16]. The UV treatment of the abutments follows the concept of photofuctionalization, the exposure of materials to intense UV light of specific wavelength, strength, and time to induce photocatalytic degradation of the material surface, which then alter its surface chemistry and energy [17,18]. The UV light irradiation of zirconia has improved human gingival fibroblast adhesion and proliferation. The increase in collagen release was also detected after 3 and 7 days of cell culture [10,17]. Thus, the photofunctionalization of the abutment could lead to improvement in soft tissue sealing ability, especially the formation of non-pocket mucosa surrounding dental abutments. Henceforth, effective healing of soft tissues prevents bacterial invasion, diminishes inflammatory changes, and elicits regeneration of gingival tissues [19,20]. The pockets and non-pocket types represent the ability of soft tissue cells to form a tight seal [21]. The biological seal from peri-implant mucosa is made up of hemidesmosomes attachment by epithelial tissue adjacent to the implant interface and from very minimal, if present, connective tissue attachment [5,13]. The significance of photofunctionalization on the bioactivity of materials for dental implant abutments becomes evident. Nevertheless, the current body of research exploring the impact of UV surface modifications on optimizing abutments for improved soft tissue contact remains quite limited. This prevailing research gap underscores the criticality and urgency of conducting further in-depth studies on the effect of UV irradiation on abutment surfaces.

The best methods to demonstrate the cell–cell reaction and attachment of peri-implant soft tissue to the materials are via histological evidence. The models that can be used to demonstrate the histological evidence are biopsies of human clinical studies [22,23,24] or animal models [3,25,26,27]. The use of 3D organotypic models fabricated in vitro has proven that this method is able to offer multiple biological endpoints for the assessment of implant-soft tissue interface [28,29,30] compared to conventional monolayer cell culture. On this account, the objective of this study was to explore the impact of UV-mediated photofunctionalization on soft tissue contour created by a three-dimensional oral mucosal model around three various types of implant-abutment materials. Specifically, the quantification of the soft tissue attachment through soft tissue contour formed at the interface of tissue and materials following photofunctionalization was performed.

## 2. Materials and Methods

### 2.1. Sample Preparation

This experiment utilized three different types of materials. All materials were prepared into discs measuring 5 mm in diameter and 3 mm in thickness by the manufacturers. These materials were (1) grade 2 commercially pure titanium (CPTi) (Edgetech Industries LLC, Tamarac, FL, USA), which acted as control material; (2) yttria-stabilized zirconia (YSZ), cut from Nacera^®^ Pearl 1 (DOCERAM Medical Ceramics GmbH, Dortmund, Germany) using Nacera^®^’s cutting tools and (3) alumina-toughened zirconia (ATZ) (Zeramex^®^ P6, Dentalpoint AG, Spreitenbach, Switzerland). Both zirconia were used as received, while silicon carbide grinding paper ranging from 1800 to 2000 grit was further used to polish the CPTi to yield standardized smooth surface roughness (S_a_) with values ranging from 0.00 to 0.5 µm/500 nm.

The samples were randomly divided into two groups and labelled either as a non-treated group (NTx) or a UV-treated group (PTx). The samples in the UV-treated group received UV light exposure for 12 min using a UV light device (Therabeam^®^ SuperOsseo, Ushio, Tokyo, Japan) (courtesy of the supplier). The device produced a combination of UV light spectra with an intensity of approximately 0.05 mW/cm^2^ (λ = 360 nm) and 2 mW/cm^2^ (λ = 250 nm). Only one disc of each material was placed in the device at a time for one experiment to standardize and optimize the exposure to all specimens. The experiments were carried out immediately after UV treatment.

### 2.2. Three-Dimensional Cell Culture and Maintenance

Ethical approval for this study was granted by the Research and Ethics Committee, Secretariat of Research and Innovation, Faculty of Medicine, Universiti Kebangsaan Malaysia (UKM PPI/111/8/JEP-2020-618). Oral epithelial cancer cell lines, TR146, and human gingival fibroblast were provided by Professor Dr. Chai Wen Lin (W.L.C.) from her previous study [13]. A media comprising Ham/F12 and supplemented with 0.5% of 5000 U/mL penicillin, 10% fetal bovine serum, and 5000 U/mL streptomycins was used in growing the TR146s. Thereafter, the cultivated cells were incubated at room temperature (37 °C) in a humidified atmosphere of 0.05 CO_2_. Gibco^®^ provided all the reagents used in the experiment (Thermo Fisher Scientific, Inc., Waltham, MA, USA). Likewise, confluency of 80% was reached before dissociating the cell growth with 5 mL of 0.25% trypsin/EDTA (Thermo Fisher Scientific, Inc., Waltham, MA, USA). The dissociated cells were then resuspended until further usage.

Patients subjected to the surgical extraction of the third molar were used as the source of human gingival fibroblasts. Healthy biopsies were collected from the patients accordingly. This step was performed by isolating the primary human gingival fibroblasts (HGFs) from the gingival biopsy, which were then cultured based on the explant method described by Chai et al. [31]. For optimal growth, the media were changed at two days intervals, and the cells were sub-cultured to passage 5 when the cells attained a confluency of 80.0%. Upon completing the culturing and passage, the HGFs were incubated at room temperature (37 °C) and humidified environment of 5% CO_2_. The HGFs were further preserved in a whole media, consisting of Dulbecco’s Modified Eagle Medium and 10% fetal bovine serum as a supplement (Gibco^®^, Thermo Fisher Scientific Pty Ltd., Scoresby, VIC, Australia), Glutamax, and Gibco^®^ Antibiotic-Antimycotic (Gibco^®^, Thermo Fisher Scientific, Inc., Waltham, MA, USA).

In this study, the construction of a 3D-OMM was achieved through modifications to a previous method utilized for implant–soft tissue interface [13]. In sum, an acellular dermal membrane (Alloderm GBR™ RTM, LifeCell Corporation, Branchburg, NJ, USA) was cut into a round shape to be inserted within a 12 mm ring insert (Corning^®^ Costar^®^ Snapwell™ Insert, Corning Life Sciences, Corning, NY, USA) in a 6-well plate. Both the HGF and TR146 cell suspensions were combined and co-cultured onto the basement membrane surface of the acellular dermis at a density of 500,000 for each cell. One ml of Ham/F12 mixture was added into the insert and incubated for approximately 2 h in the incubator to allow the cells to settle onto the membrane. Approximately 5 mL of Ham/F12 mixture was added into the wells of a 6-well plate afterward. The models were incubated as described previously, where the Ham/F12 was refreshed every two days. On Day 4 of the culture, a 4 mm perforation was made in the middle of the acellular dermal membrane, and the specimen disc was carefully inserted in the middle. Epithelial stratification was enhanced by lifting the tissues at the air–liquid interface (ALI) after culturing for 10 days. The cells were left to grow further for 14 days in the incubator while changing the media every two days. For ease of description and discussion, the 3D tissue model with materials in situ will be termed a 3D peri-implant mucosal model (3D-PIMM).

### 2.3. Soft Tissue Contour Preparation and Analyses

**Figure 1:**
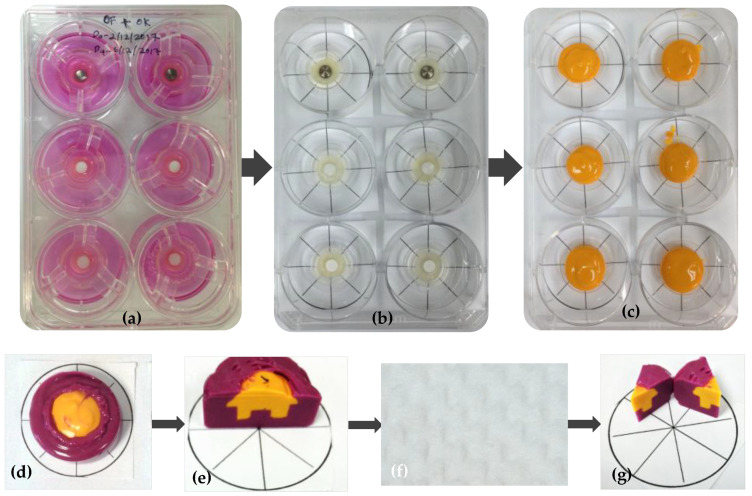
Preparation for contour analysis: (**a**) One set of experiments ready for analysis, the 3D-PIMM with material disc in situ in a 6-well plate; (**b**) Tissue models ready for impression, the ring inserts were gently dried before the injection of silicone impression material; (**c**) The yellow-coloured light-bodied silicone impression materials in situ; (**d**,**e**) a scalpel blade was used to cut the duplicated blocks of silicone models into eight portions. To ensure sections of equal sizes, the process was performed by tracing the circle drawing; (**f**) The cross-section of the middle cut, which then further divided into 8 portions as shown in (**g**).

**Figure 2:**
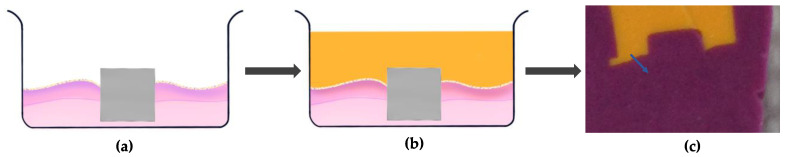
Diagrammatic presentation of the impression of soft tissue contour. (**a**) The one unit of 3D-PIMM, ready for impression procedure; (**b**) The light-bodied (yellow colour) impression material injected into the ring insert on top of the material and soft tissue surface; (**c**) Once the yellow impression material set, the specimen and tissue were carefully detached, then the regular bodied (purple colour) injected to positively duplicate the specimen. The blue arrow indicates the angle formed by soft tissue (cells) and material.

The interface contour assessment procedures were carried out on the 14th day of tissue culture. The technical procedures for impression-taking are simplified in Figure 1 and Figure 2. Two different colours of impression materials were used to present the model impression, particularly for the positive duplicate of the contour generated by the soft tissue. The ring insert and tissue model were lifted from the well and cleansed with Dulbecco’s phosphate buffer solution (Gibco^®^, Thermo Fisher Scientific, Inc., Waltham, MA, USA) three times for five minutes. Following washing, the models were gently dried by removing all excess liquid using small tip pipettes with care taken not to touch the interface between the tissue and test materials. Silicone impression materials were injected carefully into the ring inserts to form the duplicated and dried 3D-PIMM (Aquasil Ultra XLV, Dentsply Caulk International Inc., Milford, DE, USA). This procedure enables the recording of the surface and contour of the interface of the 3D-PIMM and the specimens. The impression material was allowed to solidify as per guidelines provided by the manufacturer. The impression material was allowed to be set according to the manufacturer’s instructions. Once hardened, the tissue model and specimen were separated from the impression. Following this, a purple regular-bodied silicone impression material (Examix™ NDS Monophase, GC America, Inc., Alsip, IL, USA) was injected into the hardened yellow impression materials. Subsequently, the duplicated blocks of silicone models were divided into eight portions using paper tracing, as shown in Figure 1d–g.

**Figure 3:**
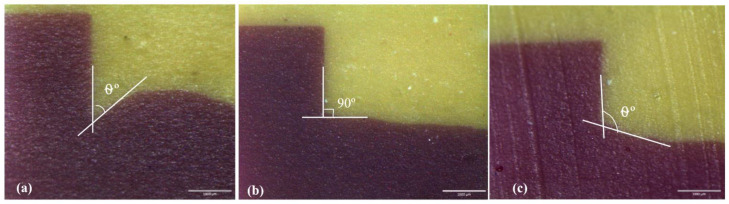
The angle between the tissue surface and the specimen disc; (**a**) score 1: θ° < 45° (**b**) score 2: 45° ≤ θ° ≤ 90° (**c**) score 3: θ° > 90°. The images were captured using a stereomicroscope.

The cut surfaces were examined under a stereomicroscope (Olympus SZ2-ILST, Olympus Corp., Tokyo, Japan). The angles were studied under imaging software (Cell^D Olympus Software, 5.1, Olympus, Tokyo, Japan). For scoring, the angles measured were categorized as θ° < 45°, 45° ≤ θ° ≤ 90°, and θ° > 90°. Figure 3 depicts the angles created at the material–soft tissue interface. The following three scores represent the categories of the angle formed at the tissue surface and the interface: (i) score 1: θ° < 45° (ii) score 2: 45° ≤ θ° ≤ 90° (iii) score 3: θ° > 90°. These scores were further categorized into pocket type for score 1: θ° < 45° and non-pocket type for score 2: 45° ≤ θ° ≤ 90° and score 3: θ° > 90°. The percentage of each score in each group was computed.

### 2.4. Assessment of Cell Morphology

For assessment of cell morphology, the specimens were carefully pulled upward from the 3D-PIMM and washed with Dulbecco’s phosphate buffer solution (Thermo Fisher Scientific, Inc., Waltham, MA, USA) three times for 5 min each to remove any loose cells. Afterward, the specimens were fixed in McDowell-Trump fixative, which was prepared in 0.1 M phosphate buffer at a pH of 7.2 at 4 °C for 24 h. The samples were prepared for scanning electron microscopy (SEM) evaluation using the hexamethyldisilazane (HMDS) technique. Briefly, after fixing, the specimens were washed with 0.1 M phosphate buffer three times for 10 min each, followed by 2 h postfix in 1% osmium tetroxide prepared in 0.2 M phosphate buffer. Following dehydration in ascending order of ethanol concentration, the specimens were immersed in an HMDS solution for 10 min. The air-dried specimens were coated with gold before viewing. Care has been taken to ensure the top surface of the discs always faces upwards during the preparation.

**Figure 4:**
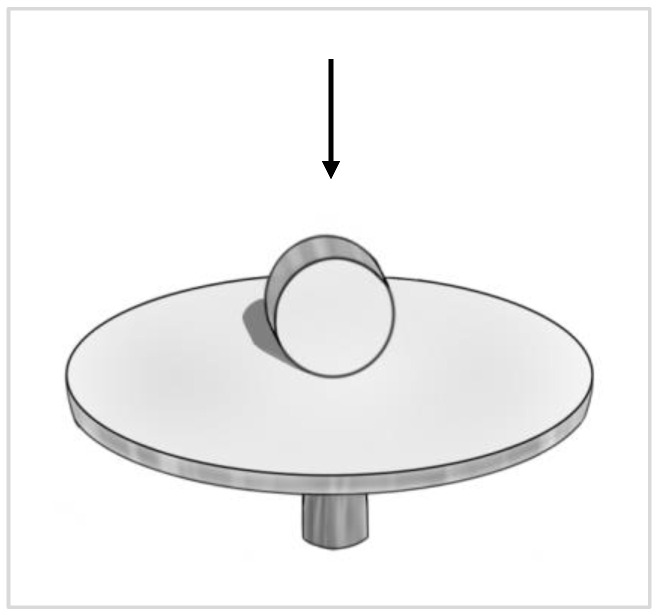
The schematic drawing of SEM pin stub with disc positioned so that the side of the disc facing the lens. The black arrow indicates the direction of SEM lens.

Scanning electron microscopy (SEM) (FEI Quanta 250 FEG SEM, Quesant Instrument Corp., Agoura Hills, CA, USA) was employed to analyze the cell morphology on all specimens. The specimens were mounted on the SEM pin stub with the side of the discs facing up in the image viewer, as shown in Figure 4.

### 2.5. Ground Section and Staining

Additionally, the 3D-PIMM units (tissue and specimens in situ) were also prepared for the ground section. The 3D-PIMM units were fixed with 4% formaldehyde buffered at pH 7.0 for at least 2 h. The models were then submerged in increasing concentrations of ethanol at 50.0%, 70.0%, 90.0%, 95.0%, and 100.0% for 60 min each in a vacuum flask. A mixture of alcohol/methylmethacrylate (MMA) resin (Technovit 7200 VLC; Kulzer, Wehrheim, Germany) at a ratio of 70:30 was allowed to pre-infiltrate into the tissue followed by infiltration of a mixture of alcohol/MMA resin at a ratio of 50:50 for one hour each. The procedures were repeated for a mixture of alcohol/MMA resin at a ratio of 30:70 for one hour and pure (100%) MMA resin for one week. Thereafter, the specimens were implanted in new epoxy resin and polymerized using a light polymerization unit for 8 h, and sectioned on a cutting machine (Exakt 300, Exakt Apparatebau, Norderstedt, Germany) using a diamond band saw (0.1 mm D32). The sections were polished on a grinding machine (Exakt 400CS, Exakt Technologies Inc., Oklahoma, OK, USA) under constant pressure and using waterproof silicon carbide papers of grit ranging from 300 to 3600 (Struers, Gothenburg, Sweden). These carbide papers assisted in generating smooth and thin sections of thickness that ranged from 30 to 50 um. The sections were stained with hematoxylin and subsequently examined under light microscopy.

### 2.6. Statistical Analysis

All the experiments were performed in triplicates. This study tested the following null hypothesis: no difference in the contour interface amongst materials regardless of UV treatment. Since the data were not numerical, the ordinal regression analysis was used to test the null hypothesis. The null hypothesis will be rejected when both the Test of Parallel Lines have *p* > 0.05 and Model Fitting Information and Parameter Estimates have *p* < 0.05.

## 3. Results

### 3.1. Contour Analyses

**Figure 5:**
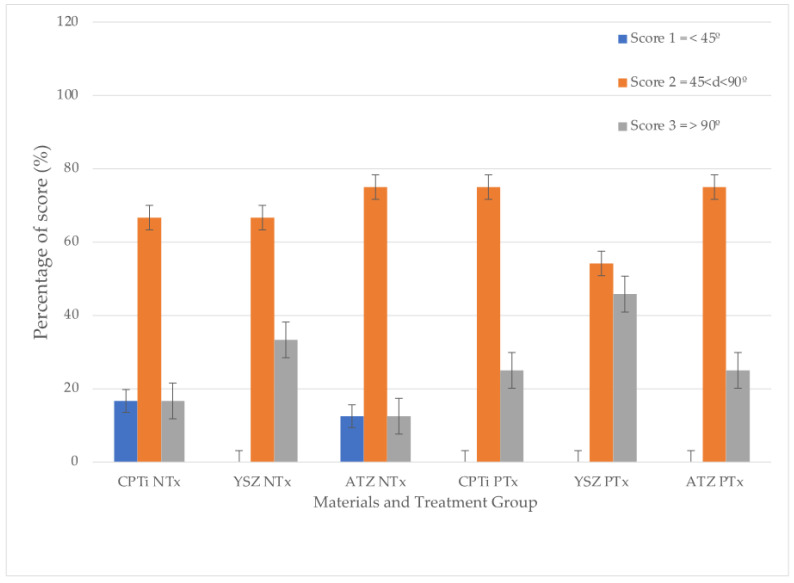
The percentage of scores for each type of material in non-treated and UV-treated groups.

The scores were tabulated in Figure 5. From the graph, it can be summarized that UV treatment on all surfaces of test materials led to the formation of a non-pocket type of contour. A statistically significant difference was observed in the contour score between the treatment groups (*p* = 0.001), indicating a tendency for lower cumulative scores for the non-treated groups. The difference in non-pocket type scores in YSZ was statistically significantly higher than the rest of the materials with *p* < 0.001, yet there was no difference between CPTi and ATZ (*p* = 0.838). The overall material-treatment effect was also significant in the formation of non-pocket type contour.

### 3.2. Cell Morphology

**Figure 6:**
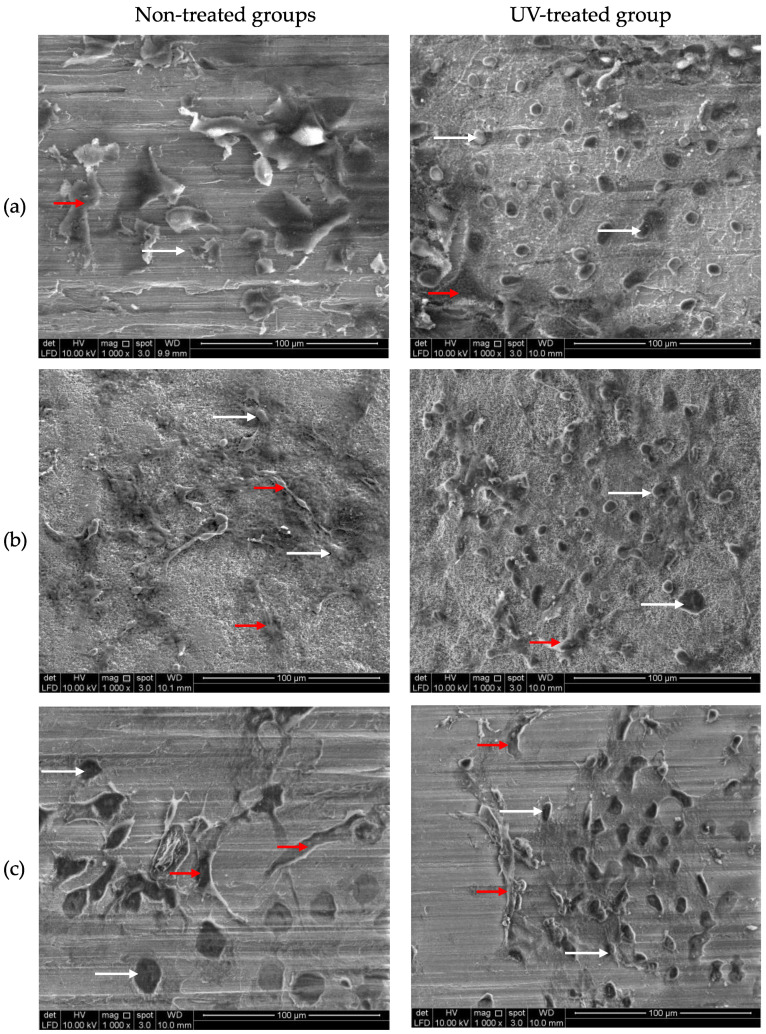
Scanning electron micrograph of non-treated specimens of each material; (**a**) CPTI, (**b**) alumina-toughened zirconia, and (**c**) yttria-stabilized zirconia groups. The distinctive features of epithelial cells and fibroblasts can still be seen in some of the micrographs (epithelial and fibroblast cells are indicated by white and red arrows, respectively).

There were higher cell numbers in the treated group when observed using SEM. Although the morphology of epithelial cells and fibroblasts are difficult to distinguish through their shapes observed through SEM, some appear distinctive in features. While the epithelial cells tend to be squamous or almost rounded with many blebs (lumps) and microvilli (small projections) showing a typical appearance of epithelial cancer cells [32], the fibroblasts are more spindle and elongated in shape (as shown in Figure 6 as white and red arrows, respectively). Both of these cells attached well to the surface, regardless of surface treatment. However, the epithelial cells appeared to attach more on the UV-treated surfaces.

### 3.3. Ground Section Analyses

**Figure 7:**
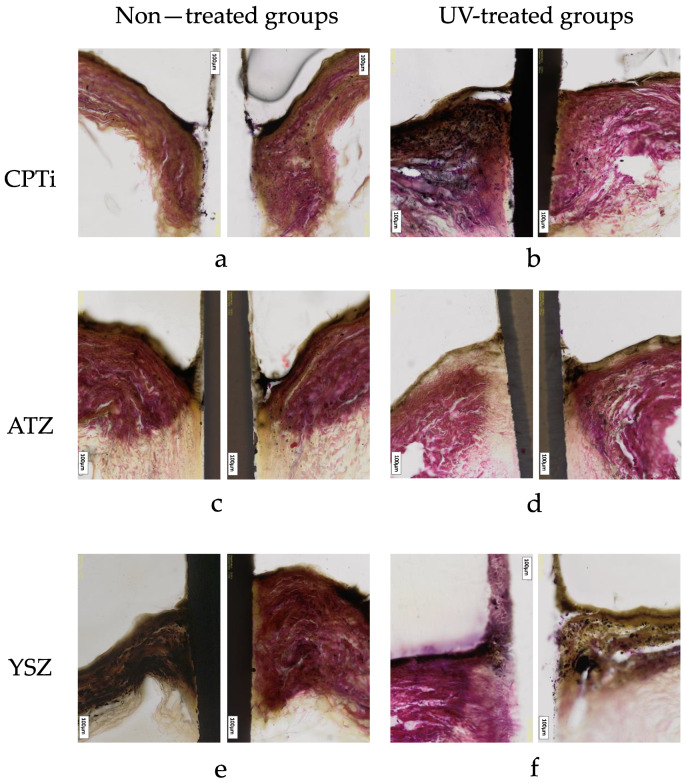
A comparison of non-treated and UV-treated ground sections of the 3D PIMMs for titanium (**a**,**b**), alumina-toughened zirconia (**c**,**d**), and yttria-stabilized zirconia (**e**,**f**) groups, respectively. The CPTi disc of the non-treated group and the YSZ disc of the UV-treated group were dislodged during the grinding process. Image (**a**,**c**) are indicative of pocket type contour, while (**b**,**d**–**f**) are categorized as non-pocket type contour. Scale bar = 100 μm.

Histologically, the ground section of the soft tissue–implant interface revealed the migration of epithelial cell attachment to the implant interface. This ground section result was observed in all materials. The attachment and the proliferation of the cells at the interface resulted in the formation of pocket and non-pocket contours of the tissue. The contour of the tissue is depicted in Figure 7.

## 4. Discussion

It is natural to find the peri-implant mucosa surrounded by a sulcus or pocket. The depth of the peri-implant sulcus is dependent upon the length of an abutment. In a healthy state, the sulcus sometimes is virtually non-existence due to the tight attachment of the mucosa to the abutment surfaces [33]. To our knowledge, this is the first study that evaluated peri-implant cells in response to UV-mediated photofunctionalization of zirconia surfaces, utilizing a three-dimensional tissue engineering technology. This study employed the contour formed by the model at the abutment interface as the endpoint for analysis. From a practical point of view, more cells migrate from the membrane, attach and proliferate on the abutment surface, reducing the depth of the pockets formed by the tissue. For a comparison, Chai et al. [31] developed a 3D oral mucosal model utilising fibroblasts and primary human oral keratinocytes. However, it was suggested that a slightly higher score for a non-pocket type in our study is attributed to the cell-line-based model’s ability to proliferate and ascend the specimen’s surface. In this study, we have used TR146, an epithelial cancer cell line derived from the neck node metastasis of buccal mucosa carcinoma. This cell is acknowledged for its ultrastructural resemblance to the normal human buccal epithelium. Additionally, this cell line is easy to culture and maintains its properties, resulting in more consistent experimental results when replicated [34,35]. Furthermore, raising the model to the air–liquid interface for a longer duration would elicit more stratifications of the epithelial cells [34,36,37], thus making the sulcus (pocket) almost becomes nonexistent. Despite exposing the models at the air–liquid interface for only four days, the pretreatment of abutments with UV has increased the formation of tissue contour of a non-pocket type category, as compared to the non-treated group. Hence, this study further verified the favourable outcomes of photofunctionalization, not only in promoting osseointegration but also in improving muco-integration.

Although studies on soft tissue reactions towards zirconia are numerous [11,17,38,39], the soft tissue reaction to zirconia under UV light’s influence is rather limited. It was discovered that the UV treatment led to more development of non-pocket types in all implant materials tested (Figure 5 and Figure 7). Additionally, it was observed that YSZ displayed more favourable results of cell attachment regardless of UV surface treatment compared to CPTi and ATZ. Despite being in the smooth surface category, the surface chemistry of YSZ was different from ATZ. This difference could be a reasonable explanation for YSZ being more favourable to soft tissue cell attachment. In the quest to enhance zirconia’s bioactivity, Yang et al. [10] investigated human gingival fibroblasts’ behaviour on zirconia disks of different surface roughness and under the influence of UV light treatment. Their study showed that UV-mediated photofunctionalization and the roughness of the zirconia surface influenced the behavior of human gingival fibroblasts and increased cell adhesion and proliferation and the release of collagen. In this study, UV surface treatment increased cellular migration from the membrane and attached to the materials. However, epithelial cells are shown to attach more to the surface compared to fibroblasts, as shown in Figure 6. In these micrographs, both epithelial and fibroblasts were distinguishable by their morphology and shape. Epithelial cells appeared squamous and flattened, while fibroblasts exhibited an elongated and spindle-shaped appearance. As observed in previous research, UV treatment of titanium enhances the adhesion of cells involved in connective tissue attachment of peri-implant mucosa, albeit with a slightly different study design compared to our study [40]. Moreover, the proliferation of human periodontal ligament fibroblasts significantly increased on the titanium dioxide (TiO_2_) coated disc following photocatalytic activity induced by 24 h of ultraviolet (UV) irradiation [41]. However, in this study, the degree of cellular proliferation of individual cells was not assessed. To provide further details, based on the findings depicted in Figure 7, the study suggests that epithelial cells exhibited a higher rate of proliferation on the UV-treated surface of the material specimens, resulting in the formation of a non-pocket-type contour.

Meanwhile, in a very recent study [42], an exposure of zirconia to excimer UV for 10 min has been shown to increase the vinculin expression of L929 fibroblasts at 6 and 24 h observations. Likewise, the experimental group exhibited enhanced expression levels of integrin β1 and collagen type I α1 compared to the control group. Based on these observations, photofunctionalization appears to be a promising technique for abutment surface modification, especially on zirconia, where physical and chemical surface modifications are rather challenging. This study is consistent with the findings of [43], even though they used a different type of cell (osteoblasts). The chemical properties of zirconia surfaces were enhanced through UV treatment, leading to improved initial cell attachment and spreading. Previous in vitro studies mostly used the fibroblasts on zirconia surfaces or the monolayer culture of keratinocytes as the basis for evaluating the interaction between implants and soft tissues [15,43,44,45]. The present 3D model experiment depicted more clinically relevant findings from the oral mucosa model at multiple-endpoint analyses of peri-implant tissues. The results provided more information relative to the monolayer cell culture systems. This technique was novel, straightforward, and easy to conduct for the evaluation of soft tissue surrounding the peri-implant mucosa. The combination of these techniques facilitated quantitative biological analyses of how the dimension of the peri-implant tissue is affected by modifying the material’s surface [28,29]. This present study showed that the 3D oral mucosal model could be used to evaluate the efficacy of UV photofunctionalization in improving the cellular attachment of the zirconia abutment of an implant. Not only is this 3D model useful for assessing the toxicity of biomaterials prior to animal studies, but it also provides more meaningful clinical translation than 2D or monolayer studies [29,46]. Among the endpoint outcomes that can be evaluated from this model are cytotoxicity or cell viability assays [37,47], ELISA test to quantify the release of proinflammatory cytokines [46], and histology assessment to visualize the epithelial or connective tissue morphology [48]. These 3D oral mucosal models have been compared to a 2D monolayer for the biological evaluation of glass ionomer cement and ethanol-containing solution [48,49]. Both studies have proved that the monolayer keratinocytes or fibroblasts are more sensitive to the tested material compared to the 3D oral mucosal models.

Nevertheless, this study presents several limitations. The procedure to duplicate the 3D-OMM with materials in situ posed several challenges. Due to the delicate nature of the 3D-PIMM, the impression-taking technique performed in this study and the act of removing the fluid from the model may affect the contour/attachment formed by the cell at the interface of materials and soft tissue. The pressure generated by these materials has the potential to compress and, therefore, distort the contour and attachment. Additionally, the impression materials injected into the model may not flow well into the crevice of the material–soft tissue interface due to the hydrophobic nature of the impression materials if the model is not dried enough. To overcome this limitation, the use of intra-oral scanners may produce more accurate digital impressions [50] and able to reduce imperfection [51].

Secondly, the 3D-PIMM developed in this study was based on fibroblasts and keratinocyte cell lines only rather than normal primary gingival keratinocytes and fibroblasts. To mimic the oral condition, the primary gingival cells should be used. The 3D-PIMM developed in this study consisted of the connective tissue collagen layer (made from the acellular dermal matrix), human gingival fibroblasts, and multiple layers of epithelial cells. However, it is yet to be determined which cells exhibit a stronger inclination for migration, adhesion, and attachment to the material surface. On this account, the ascertainment of the specific type of cells that remain attached to various abutment surfaces after conducting the pull test would be the focus to further explore the effect of photofunctionalization on abutment surfaces, and the nature of the soft tissue attachment should be conducted. To accomplish this, the method involves conducting double immunocytochemistry staining for both human gingival fibroblast and oral epithelium in one specimen; for example, vimentin or α-SMA can be used to stain human gingival fibroblasts, and pancytokeratin or cytokeratin 14 for epithelial cells and gene markers [52,53]. In the same manner, double immunohistochemical staining can also be performed on the histological ground section of the soft tissue–implant interface in addition to the hematoxylin and eosin stain.

## 5. Conclusions

Considering the limitations of this study, one can draw the conclusion that the UV-mediated photofunctionalization has improved the peri-implant region’s soft tissue form with the formation of non-pocket type contour evaluated using a three-dimensional peri-implant mucosal model. Zirconia (YSZ) formed a better soft tissue contour than ATZ and titanium. Moreover, the 3D-PIMM presents boundless opportunities and reliability for assessing biological endpoints of peri-implant soft tissue, particularly in evaluating abutment surface modification to enhance connective tissue attachment. It could serve as an alternative to animal experiments in preclinical studies, thus reducing the need for animal testing.

## Data Availability

The data presented in this study are available on request from the corresponding authors.
